# Exploring the Potential of Bee-Derived Antioxidants for Maintaining Oral Hygiene and Dental Health: A Comprehensive Review

**DOI:** 10.3390/antiox12071452

**Published:** 2023-07-19

**Authors:** Poonam Choudhary, Surya Tushir, Manju Bala, Sanjula Sharma, Manjeet Kaur Sangha, Heena Rani, Nileshwari Raju Yewle, Parminder Kumar, Diksha Singla, Deepak Chandran, Manoj Kumar, Mohamed Mekhemar

**Affiliations:** 1 Department of Agricultural Structures and Environment Control, ICAR-Central Institute of Post-Harvest Engineering and Technology, Ludhiana 141004, India; poonam@icar.gov.in; 2 Department of Food Grain and Oilseed Processing, ICAR-Central Institute of Post-Harvest Engineering and Technology, Ludhiana 141004, India; manju.bala1@icar.gov.in; 3 Oilseeds Section, Department of Plant Breeding and Genetics, Punjab Agricultural University, Ludhiana 141004, India; drsanjula@pau.edu; 4 Department of Biochemistry, College of Basic Sciences and Humanities, Punjab Agricultural University, Ludhiana 141004, India; manjeet_kaursangha@pau.edu; 5 Department of Biochemistry, Punjab Agricultural University, Ludhiana 141004, India; heena-1860003@pau.edu (H.R.); singladiksha3@gmail.com (D.S.); 6 Department of Entomology, Purdue University, West Lafayette, IN 47907, USA; nyewle@purdue.edu; 7 Department of Soil Science, Punjab Agricultural University, Ludhiana 141004, India; pk2704@pau.edu; 8 Department of Animal Husbandry, Government of Kerala, Palakkad 679335, India; drdeepakchandran24@gmail.com; 9 Chemical and Biochemical Processing Division, ICAR-Central Institute for Research on Cotton Technology, Mumbai 400019, India; 10 Clinic for Conservative Dentistry and Periodontology, School of Dental Medicine, Christian-Albrecht’s University, 24105 Kiel, Germany

**Keywords:** bee products, honey, propolis, royal jelly, oral care, bioactivities, oral pathology

## Abstract

Honey bee products comprise various compounds, including honey, propolis, royal jelly, bee pollen, bee wax and bee venom, which have long been recognized for their pharmacological and health-promoting benefits. Scientists have discovered that periodontal disorders stem from dental biofilm, an inflammatory response to bacterial overgrowth produced by dysbiosis in the oral microbiome. The bee products have been investigated for their role in prevention of oral diseases, which are attributed to a myriad of biologically active compounds including flavonoids (pinocembrin, catechin, caffeic acid phenethyl ester (CAPE) and galangin), phenolic acids (hydroxybenzoic acid, hydroxycinnamic acid, p-coumaric, ellagic, caffeic and ferulic acids) and terpenoids. This review aims to update the current understanding of role of selected bee products, namely, honey, propolis and royal jelly, in preventing oral diseases as well as their potential biological activities and mechanism of action in relation to oral health have been discussed. Furthermore, the safety of incorporation of bee products is also critically discussed. To summarize, bee products could potentially serve as a therapy option for people suffering from a variety of oral disorders.

## 1. Introduction

Honey bees are members of the genus Apis, which means “bee” in Latin. The prefix “api” is frequently used in beekeeping terms such as apiarist or a beekeeper. Apitherapy is defined as the use of beehive produces such as honey, pollen, royal jelly, propolis, bee venom and wax for treating and healing of ailments as well as in boosting the human immune system [1,2]. The origin of apitherapy can be pinpointed to more than 6000 years back in medicine of ancient Egypt [3]. The bee products are rich in natural antioxidants, and these are commonly used as natural remedies for health maintenance in traditional medicine in many countries. Honey bee products are popular among people of all ages, and are used across cultural and ethnic boundaries, and all religious and cultural beliefs promote and embrace the usage of honey bee products [4]. Among the various bee products, honey, propolis, and royal jelly have been discussed for improving oral health in the present review.

Natural honey is the world’s oldest sweetener, with records indicating that it was used around the planet several million years ago [5]. Honey is produced by the honey bees that collect nectar from flowers and process it through repeated digestion and regurgitation. It comprises fructose (38.5%), glucose (31%), other sugars (12.9%), water (17.1%), protein (0.5%) and minerals such as Ca, Co, Fe, Mg, Mn, Zn, Na, F and K [6,7]. In addition, it comprises valuable bioactive compounds, such as phenolic acids, carotenoids, flavonoids, organic acids, ascorbic acid, enzymes and other proteins [8,9,10,11].

Propolis, also known as “bee glue”, is the third most important bee product after honey and wax. The term “propolis” comes from the Greek words “pro” for defense and “polis” for city or community [12]. Its complex composition includes phenolic compounds (58%), beeswax (24%), lipids and wax (8%), flavonoids (6%), terpenes (0.5%), bio-elements (0.5%) and other substances (3%) [13,14]. It provides thermal insulation and protects the hive from invaders and is used to strengthen the hive by filling cracks and holes [13,15,16].

Royal jelly is a white, viscous jelly-like material secreted by worker bees’ hypopharyngeal and mandibular glands [17]. Royal jelly consists of water (50–60%), proteins (18%), carbohydrates (15%), lipids (3–6%), mineral salts (1.5%) and vitamins [18,19,20]. With around 185 organic compounds, royalactin is one of the major royal jelly proteins (MRJPs) [18,19,20]. Besides polyphenolic and flavonoid compounds, royal jelly is rich in essential amino acids, small peptides, fatty acids and vitamins. Other prominent carboxylic acids discovered in royal jelly include sebacic acid (SA) and 10-hydroxy-2-decenoic acid (10H2DA), which has not been recorded in any other natural raw material or even in any other apiculture product [21].

According to a report, oral diseases are becoming a matter of concern globally [22]. Oral health, in turn, affects the systemic health. An increased risk of cardiovascular diseases, digestive problems, diabetes and bacterial pneumonia has been reported in patients with poor oral health [23]. Various studies have explained the unique bioactive components of honey bee products that confer therapeutic and medicinal properties and improve the overall health. The bioactive compounds found in honey, propolis and royal jelly are known to possess antioxidant, anti-microbial, anti-inflammatory, anti-cancer, anti-ulcer, immunomodulatory, neuromodulatory and metabolic syndrome preventing activities [24,25,26,27,28,29,30,31,32,33,34,35,36]. Due to these properties, these products are being used/or show wide applications in preventing oral diseases like stomatitis, mouth ulcers, dental caries, plaque, gingivitis, periodontitis, cavity infection, dentin hypersensitivity, oral cancer, malodor and mucositis [15,37,38,39,40].

**Figure 1:**
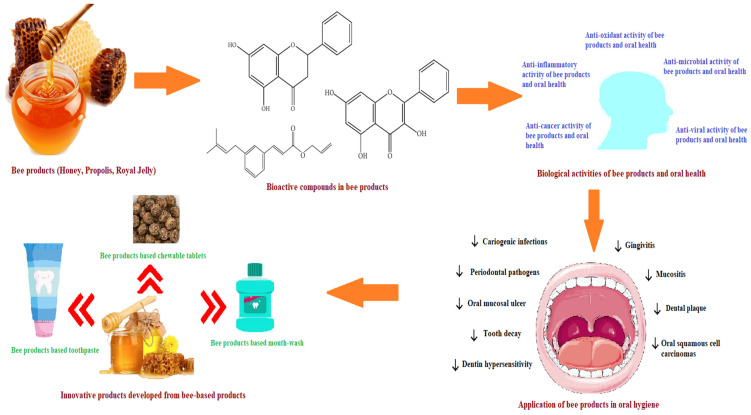
Various components discussed in current review.

In today’s health-conscious society, honey bee products are gaining attention in traditional and modern medicine [41]. Application of bee products alleviates the consequences of oral disorders in a cost-effective manner. Thus, it is a challenge for researchers all over the world to make their effective use and realize their maximum medicinal benefits. There are no critical miscellanea on key facts about the role of bee products in treatment of oral diseases. As a result, the focus of this review will be on the major bioactive components, possible pharmacological characteristics and mechanism of action of bee products in the treatment of oral diseases. Figure 1 depicts the various components as discussed in this review.

## 2. Bioactive Compounds of Bee Products in Relation to Oral Health

Natural bee products (honey, propolis and royal jelly) are generally considered as high-quality sources of bioactive compounds, having impressive biomedical potential. The therapeutic effect of these products is associated with their bioactive composition and plant source from which they are derived [42].

### Bioactive Compounds

Honey is rich in bioactive compounds such as phenolic acids and flavonoids, which contribute to its beneficial properties [8,9,10,43]. The composition of these compounds in honey can vary based on factors such as environmental conditions, geographical location, production process, and the specific flora from which the honey bees collect nectar [10,15,43,44]. Dark-colored honey is believed to contain higher levels of bioactive chemicals compared to light-colored honey. For example, manuka honey has been found to contain compounds such as pinocembrin, chrysin, pinobanksin, kaempferol, luteolin, isorhamnetin, galangin, sakuranetin, quercetin and magniferolic acid [45]. Similarly, honey produced from different plant sources, such as *Echium plantagineum* L., may exhibit variations in their phenolic acid and flavonoid content [46]. Flavonoids are another significant group of bioactive components found in honey. Their content can vary among different honey types, with values ranging from 1.93 to 21.16 mg of quercetin equivalents per 100 g in samples such as sunflower and Heather honey [47]. Flavonoids possess antioxidant properties and contribute to protecting cell membranes by reducing lipid peroxidation and scavenging free radicals [48,49]. The composition of flavonoids in honey is influenced by floral origin and geographical location. For example, chrysin and apigenin are commonly found flavonoids in honey from Spain and New Zealand, while orange blossom honey has a significant amount of quercetin [50]. Propolis, another bee-derived product, contains bioactive compounds such as flavonoids (apigenin, acacetin, chrysin, catechin, naringenin, luteolin, galangin, kaempferol, rutin, pinocembrin, myricetin and quercetin) and phenolic acids (cinnamic acid and caffeic acid) [51,52,53]. The composition of propolis varies depending on its geographical and botanical origins [54]. Cinnamic acid derivatives and flavonoids, collectively known as citrin and vitamin P, are considered prime bioactive compounds in propolis [4,55]. The specific composition of propolis differs among regions, with caffeic acid phenethyl ester (CAPE) being the most common compound in temperate propolis, geranyl flavanones prevalent in Pacific and African propolis, and prenylated phenyl propanoids, acetophenones, terpenoids and aromatic acid derivatives found in tropical regions [4,55].

Royal jelly is composed of a small amount of minor bioactive compounds, including flavonoids such as flavanones (hesperetin, naringenin, isosakuranetin), flavones (chrysin, acacetin, luteolin, epigenin and its glycoside), flavonols (kaempferol and isorhamnetin glycosides) and isoflavonoids (coumestrol, genistein, formononetin) [18,56,57]. Phenolic acids such as octanoic acid, dodecanoic acid, pinobanksin and 1,2-benzenedicarboxylic acid, along with their esters, are also present in royal jelly [18,56]. The concentration of these phenolic acids and flavonoids in royal jelly is influenced by several factors, including the type of plants used by bees, seasonal variations and environmental factors [58,59]. In a study by Nagai and Inoue [60], the total phenolic compound content in royal jelly powder was reported as 21.2 µg/mg in water and 22.8 µg/mg in an alkaline extract. Different bioactive compounds in bee products and their actions are represented in Table 1.

## 3. Biological Activities of Bee Products in Relation to Oral Health

### 3.1. Antioxidant Activity of Bee Products and Its Effects on Diseases of the Oral Cavity

Honey has been reported to show antioxidant activity since the phenolic level is related to radical scavenging activity of honey and other bee products [64,65,66]. Polyphenols are regarded to be the key components responsible for honey and bee products’ antioxidant properties. These components have the ability to counteract the effects of oxidative stress, which is a key factor in the development of many diseases. [67]. In the area of dental medicine, the awareness of the potential benefits of bee products as antioxidants is rising. During normal metabolism, reactive oxygen species (ROS) are generated, which include free radicals (superoxide anion radical, hydroxyl radical, etc.) and non-radical molecules (hydrogen peroxide, singlet oxygen, lipoperoxides, etc.). These ROS interact with lipids, protein components in the cell membranes, enzymes and DNA of cell and may cause damage to them, which, in turn, may lead to various diseases. The antioxidants are compounds that seize free radicals before they can cause damage to the cell as they donate hydrogen atoms from their hydroxyl groups to free radicals [68]. According to Zhang et al. [32], polyphenols have the capability to control the nuclear factor kappa B (NF-κB), which is associated with modulating cell signaling pathways involved in cancer and inflammation. Antioxidants are generally given as oral ingestion, diet or vitamin supplements and in the form of nutraceuticals. Natural antioxidants that can be applied topically such as mouth rinse, gel, paste, gum, or lozenge compositions are nowadays gaining importance. These topical antioxidants may help to reduce ROS, which may act as inflammatory factors in the development of gingival and periodontal problems [69].

Honey has a high level of antioxidant activity and can lessen the effects of oxidative reactions that produce free radicals. Buckwheat and Heather honey are typically dark brown or black in color and are the richest sources of antioxidants and can ameliorate oxidative stress [70,71] while manuka honey has been reported to be rich source of flavonoids and also possesses higher antioxidant properties in comparison to other types of honey. Kishore et al. [72] compared antioxidant activity of Tualang, Indian forest, pineapple and Gelam honey samples and reported highest antioxidants in Tualang honey collected from Tulang honey bees in forests of Malaysia. Rosa et al. [73] studied TPC and antioxidant activities in Asphodel, Acacia, Eucalyptus, Heather, Citrus, Honeydew and Strawberry tree honey samples and found that Strawberry tree honey expressed the highest 2,2-diphenylpicrylhydrazyl (DPPH) and ferric reducing ability of plasma (FRAP) activities. They further reported 2,5-dihydroxyphenylacetic acid and homogentisic acid (HGA), major phenolic compound and a chemical marker for strawberry tree honey, were the potent antioxidants. Alvarez–Suarez et al. [74] reported significant radical scavenging activity in the ether-soluble phenolic extracts of Cuban honeys. Yuslianti et al. [75] used Rambutan honey to investigate free radical scavenging capacity (in vitro) and lipid peroxidation inhibition of oral mucosal wounds (in vivo) in male Wistar rats. They reported that 1 mg/mL honey showed 45.3% DPPH inhibition. Significant (*p* = 0.028) reduction in lipid peroxidation in oral mucosa wound tissue was observed. Toczewska et al. [76] reported that the antioxidant capacity in gingival crevicular fluid and saliva of periodontitis patients is diminished as a result of the chronic inflammatory process, which further makes proteins, lipids and DNA prone to oxidative damage and may result in the progressive devastation of the periodontal attachment apparatus. The beneficial effect of honey rich in polyphenolic compounds in enhancing the antioxidant capacity of oral fluids has been reviewed [77]. According to Ding et al. [78], the antioxidant properties of polyphenols appear to give significant protection against oral malignancies. Phenolic chemicals aid in the treatment of periodontal disease, the strengthening of teeth and the prevention of tooth decay [79,80,81]. Patients with recurrent aphthous stomatitis (RAS) exhibited considerably lower salivary antioxidant levels than healthy controls, according to Babaee et al. [82]. They correlated that ulcerations created ROS, which resulted in a reduction in antioxidant compounds in the mouth. Honey, when applied topically, has antioxidant components that can help minimize the effects of ROS activity during the development of ulcers.

Propolis possesses high concentration of phenolics [83] and is well-known for its antioxidant and radical-scavenging properties [61,81]. Bioactive substances such as pinocembrin, chrysin and pinobanksin have high antioxidant and anti-radical properties [82]. In DPPH and ORAC tests, pinobanksin-3-acetate was found to be the most powerful antioxidant component [84]. Fabris et al. [85] studied ethanol extracts of Russian and Italian propolis and found similarity in their total phenol content and antioxidant activity while low levels of phenolics and antioxidants were observed in ethanolic extract of Brazilian propolis. Zhang et al. [32] reported that antioxidant activity in Brazilian green propolis was due to 4,5-dicaffeoylquinic acid, 3,4,5-tricaffeoylquinic acid, artepillin C compounds and 3,5-dicaffeoylquinic acid. Total polyphenol and total flavonoid levels in poplar propolis contribute to antioxidant action [86]. In vitro investigations by Kumari et al. [87] and Bonamigo et al. [88] reported that propolis extracts were discovered to have antioxidant properties similar to the synthetic antioxidants, butylated hydroxytoluene or ascorbic acid. The total phenolic content (TPC), antioxidant activity and free radical scavenging activities (FRSA) of 70 Turkish samples of various honey bee products (honey, pollen, royal jelly and propolis) and their mixtures were examined by Ozkök and Silici [89]. Among the studied samples, honey bee propolis exhibited highest antioxidant and FRSA activity. Moreover, TPC was also found to have a positive relationship with antioxidant activity and FRSA. Kocot et al. [90] evaluated the total phenolic content (30 to 200 mg gallic acid equivalents/g DW), DPPH free radical-scavenging activity (20 to 190 g/mL) and flavonoid content (30 to 70 mg quercetin equivalents/g) of several propolis extracts. Aghel et al. [91] studied the useful result of propolis antioxidants on saliva in the test animals. El-Sharkawy [92] used propolis as a dietary supplement and found that when compared to the control group, the propolis-treated group had a substantial decrease in pocket depth (PD) and a rise in clinical attachment level (CAL), which could be due to antioxidant activity and other propolis properties. Giammarinaro et al. [93] compared the propolis efficacy with chlorhexidine in 40 patients of gingivitis and found that propolis-treated patients had improved results in terms of oxidative stress markers in their saliva, as well as significant improvements in their periodontal health. CAPE, extracted from propolis, is a strong antioxidant compound that is potentially used in oral squamous cell carcinoma (OSCC) patients for adjuvant therapy [38].

Royal jelly has been found to have antioxidant action owing to its total phenolic content, fatty acids and proteins. The small peptides contained in royal jelly, which are made up of 2–4 amino acid residues, have a high antioxidant activity. Tyrosine residues at the C-terminus of most active peptides in royal jelly allow them to scavenge hydroxyl radicals and H_2_O_2_ [94]. Nagai and Inoue [60] investigated the antioxidative activity and scavenging ability of protein fractions of royal jelly against free radicals such as the DPPH (1,1-diphenyl-2-picrylhydrazyl) radical, hydroxyl radical and superoxide anion radical. The antioxidant effect of royal jelly was studied by Silici et al. [95], who found a decrease in malondialdehyde levels and an increase in catalase superoxide and glutathione peroxidase dismutase activities. Park et al. [96] studied the assays of purified recombinant *Apis mellifera* major royal jelly proteins (AmMRJPs) and found that these proteins displayed radical-scavenging activity with 1,1-diphenyl-2-picrylhydrazyl and protection against oxidative DNA damage. Effect of the harvest time and the initial larval age has significant effect on the antioxidant potential in Royal jelly. Royal jelly collected 24 h after the larval transfer had the highest antioxidant activity in terms of DPPH radicals, prevention of linoleic acid peroxidation and reducing power [97]. Anatolian royal jelly samples were examined by Kolayli et al. [21] for their chemical composition and antioxidant capabilities. The total phenolic content ranged from 91.0 to 301.0 mg gallic acid equivalents/kg fresh weight. Anatolian royal jelly samples were found to be similar with other royal jelly samples around the world. Balkanska et al. [98] evaluated the antioxidant activity of royal jelly (RJ) collected from different areas of Bulgaria. The FRAP and total polyphenols showed variability and values ranged from 0.44–8.49 mM Fe^2+^/g and from 11.66–36.73 (mgGAE/g) for FRAP and total polyphenols, respectively. Uçar and Barlak [99] studied the antioxidant activity of water, dimethyl sulfoxide (DMSO) and methanol extracts of royal jelly from Bursa province in Turkey and found all the extracts showed total phenol content (TPC) and free radical scavenging capacity. The Table 2 represents the different biological activities of bee products and their relation in improving the oral health.

### 3.2. Anti-Microbial Activity of Bee-Based Products and Its Effects on Diseases of the Oral Cavity

The microbial flora associated with oral disease is diverse, including aerobic and anaerobic bacteria, viruses, parasites and other pathogens [113,114]. Oral microbial infections have been treated with a variety of agents, like amine fluorides, chlorhexidine, cetylpyridinium chloride and ethanol, which are commonly present in mouthwashes. But these have been proven to discolor teeth and may be toxic and cause oral malignancies, as well as have an undesirable taste [115]. Furthermore, the interest in such natural product as a possible source of novel antimicrobials has grown due to a concurrent decline in the number of effective antibiotics available and the ever-increasing prevalence of antibiotic resistance among pathogenic bacteria [116,117]. Natural substances discovered in bee-based product such as honey, propolis and royal jelly have been used for its anti-bacterial, anti-fungal and antiviral activities as well as anti-microbial benefits [118]. The antimicrobial properties of propolis against oral pathogens are attributed to the flavonone pinocembrin, amyrins, flavonol galangin and the caffeic acid phenethyl ester by inhibiting the bacterial RNA polymerase [119].

#### 3.2.1. Anti-Bacterial Activity of Bee-Based Products and Its Effects on Diseases of the Oral Cavity

Numerous bacterial microbiotas inhabit in the mouth cavity with *Streptococcus mutans, Porphyromonas gingivalis, Staphylococcus* and *Lactobacillus* being the common oral bacteria [120]. *Lactobacillus* is a bacterium that produces lactic acid by fermenting sugar, which can easily cause caries [121]. *P. gingivalis* is a periodontal pathogen that is a Gram-negative anaerobic bacterium of non-glycolytic nature. *P. gingivalis*, if left untreated, could cause the teeth to fall off from gums. Further, anti-bacterial resistance has become a major problem globally that has prompted scientist community to study active ingredients used before the antibiotic era. Van Ketel, a Dutch scientist, was the first to identify honey’s bactericidal properties in 1892 [122]. This anti-bacterial activity is attributed to its properties which include: (i) its hygroscopic nature that can draw moisture out of the environment and dehydrate bacteria, (ii) its high sugar content and acidity (low pH) that prevents the microbes from growth [123], (iii) presence of hydrogen peroxide and phenolic acids [124], flavonoids [125] and lysozyme [126] as oxidizing agents restrict the bacterial responses to proliferative signals due to which bacterial growth remains arrested [127], (iv) phytochemical components like methylglyoxal (MGO) (non-peroxide) trigger modifications in the shape of bacterial flagella and fimbriae that impede bacterial adhesion and motility [128,129] and (v) an anti-microbial peptide, bee defensin-1 [130]. Honey’s pH (3.2–4.5) is low enough to suppress specific bacterial infections, such as *Salmonella* spp. (4.0), *E. coli* (4.3), *P. aeruginosa* (4.4) and *S. pyogenes* (4.5) [131], and enhances the healing process of wound through epithelialization [132]. Moreover, its anti-bacterial action was not shown to be affected by loss of its acidity after dilution [133]. Shiga et al. [134] reported the antibacterial action of honey was derived from methylglyoxal component, which help in oral disorders like halitosis (a bad breath condition). Honey is also used to prevent dental plaque, gingivitis, mouth ulcers and periodontitis. Honey may decrease dental plaque production and aid in reducing gingivitis associated with orthodontic operations, according to a study conducted by Patel et al. [135] on bacterial isolates collected from patients receiving orthodontic treatment. Honey inhibits anaerobic bacteria, which helps to prevent periodontal disease caused by *Porphyromonas gingivalis* [136]. In a trial of 150 dyspeptic individuals, honey consumption at least once a week dramatically reduced the chance of *Helicobacter pylori* infection. [137]. Honey can be used to replace glucose in oral rehydration, and its anti-bacterial qualities helped to shorten the duration of bacterial diarrhea [130].

The anti-bacterial properties of propolis have also been reported due to presence of caffeic acids, benzophenone derivatives, ferulic acid, prenylated coumaric acid and diterpenic acids [12,138,139,140,141]. The anti-bacterial properties of propolis have been shown to prevent the formation of bacterial plaques [142]. When compared to chlorhexidine, propolis solutions had a lesser cytotoxic effect on human gum fibroblasts. Mouthwashes containing propolis were discovered to have anti-bacterial action towards *S. mutans* and has been used as an alternative treatment for dental caries prevention [143]. Propolis fluoride was administered to teeth of children with dental caries surface to investigate the efficacy of propolis in combating dental caries. In another study, researchers treated individuals with a 10% hydroalcoholic solution of propolis extract, who were suffering from chronic periodontitis, and discovered a 95% reduction in chronic periodontitis [144]. It played an important role in the repair of tooth pulp [145]. The combined use of mouthwash and toothpaste with ethanolic extract of propolis improved the prevention of microbial infection and the treatment of gum inflammation [15,146].

The protein components of royal jelly like RJ proteins, royalisin, jellenies and enzymes, such as glucose oxidase, have significant anti-microbial potential [147]. The fatty acid 10-HDA, which also has immunomodulatory effects, is responsible for royal jelly’s anti-bacterial capabilities [148]. Terada et al. [149] found that the fatty acids contained 32% (10-HDA), 24% gluconic acid, 22% 10-hydroxydecanoic acid (HDAA), 5% dicarboxylic acids and several other acids, which effectively suppressed oral pathogens *S. viridans* and *S. mutans* as well as *S. aureus* and *S. epidermis*. Overall, royal jelly is a natural product having minimal side effects that works in synergy with other anti-plaque agents. Therefore, it can be used as an alternative to synthetic antibiotics [150].

#### 3.2.2. Anti-Fungal Activity of Bee-Based Products and Its Effects on Diseases of the Oral Cavity

In human mouth, around 85 different fungi could be found and the most significant one is *Candida* [151]. When the oral microbiota is balanced, Candida sp. remains neutral; nevertheless, if the equilibrium is disrupted, this fungus will seek a chance to harm oral health. The *Candida* species, particularly *Candida albicans,* are major human fungal pathogens that can cause superficial oral mucosal infections often known as Candidiasis. According to reports, honey, propolis and royal jelly have anti-fungal properties against *Candida albicans*. Honey is also effective against dermatophytes (*Microsporum ferrugineum*, *Trichophyton mentagrophyte*, *Trichophyton longfeuseus*, *Trichophyton semmie*, *Trichophyton tonsurance*), parasitic fungi (*Allescheria boydii*), saprophytes (*Mucor mucaralis*) and *Aspergillus* species, according to Sheikh et al. [152]. Honey’s anti-fungal action has been associated with: (i) H_2_O_2_, which is formed in honey after dilution as a result of glucose oxidation [153,154], (ii) MGO, bee defensin-1 and other bee substances (e.g., phenolic compounds and flavonoids of floral origin and lysozyme), and (iii) honey’s osmotic impact. The honey’s mode of action, which inhibits biofilm development or accelerates the rupture of mature biofilms, includes the breakdown of cell membrane integrity and suppression of extracellular polysaccharide matrix creation [155]. Honey could be used to treat oral candidiasis so as to prevent infections from becoming more severe. In pilot research conducted by English et al. [156], patients were given chewable ‘honey leather’ and showed a substantial reduction in mean plaque scores and bleeding sites; the same strategy could be used to treat oral candidiasis. The flavonoid content of propolis confers its anti-fungal properties and also prevents division of fungal cell that further disrupts fungal cell wall and cytoplasm and which is comparable to several antibiotics [157]. Geopropolis produced by bees such as *M. fasciculata* possesses anti-fungal characteristics, according to Feres et al. [158]. In recent in vivo studies, geopropolis produced by *M. fasciculata* was reported to lower salivary *S. mutans* populations. This anti-fungal effect is attributable to caffeic acid derivatives (benzyl ester and pterostilbene) and flavonoids (sakuranetin, pinobanksin and pinocembrin) found in bee propolis. Propolis extract applied topically to oral *Candida albicans* lesions achieved remission in three weeks, with treatment efficacy comparable to nystatin, the most commonly used anti-fungal medication [159]. Royalisin is an insect defensin extracted from the royal jelly (RJ) and its inhibitory effect against a large spectrum of fungi was observed [160,161,162]. Royalisin has also been shown to have anti-fungal effect against *Botrytis cinerea* [163]. According to Melliou and Chinou [164], royal jelly carboxylic acids such as sebacic acid have high anti-fungal action against *Candida albicans*, *Candida tropicalis* and *Candida glabrata.*

#### 3.2.3. Antiviral Activity of Bee-Based Products and Its Effects on Diseases of the Oral Cavity

The oral microbiome also contains viruses, primarily phages [165]. When human body is acquainted with certain diseases, other certain viruses may also arise in the mouth, for example, mumps virus [166] and HIV [167] are the most frequent. The DNA and RNA based viruses are responsible for oral cavity infection in the mucosal epithelium, which may further lead to ulceration or blistering [168]. Human herpes virus (HHV) members including varicella-zoster virus (VZV), HSV-1 and 2, HHV-6, HHV-7, and HHV-8, cytomegalovirus (CMV), human papillomaviruses (HPV) and Epstein-Barr virus (EBV) cause primary oral infections and diseases such as herpes ulcers, tumors, herpes zoster, precancerous lesions, periodontitis and herpes chicken pox etc. The oral mucosa may also be affected by secondary pathological processes [169]. Honey’s efficacy in the treatment of recurrent HSV lesions and oral mucositis in cancer patients has been well documented [30,170,171]. Antiviral effects of propolis have been established against a wide spectrum of viruses. Pagani [172] studied the antiviral activity of propolis flavonoids such as acacetin, kaempferol, chrysin, quercetin and galangin against diverse types of adenoviruses, influenza viruses, rotavirus, herpesvirus and coronavirus. Serkedjieva et al. [173] found that propolis phenolics, particularly isopentyl ferulate, have potent antiviral properties against the H3N2 influenza A virus. It is often applied in the treatment of diseases involving the oral cavity and gums. Antiviral effects of propolis against herpes viruses are also promising. In vitro studies have also revealed that the flavonoids present in propolis inhibited herpes virus strains from replicating intracellularly [174]. Using the plaque assay approach, in an extra-somatic environment, Hashemipour et al. [175] studied the antiviral properties of honey, royal jelly and acyclovir on herpes simplex virus-1. Honey, royal jelly and acyclovir at concentrations of 500, 250 and 100 g/mL, respectively, had the strongest inhibitory effects on HSV-1.

#### 3.2.4. Anti-Inflammatory Activity of Honey Bee-Derived Products and Its Effects on Diseases of the Oral Cavity

Inflammation is the immune system’s natural innate response to infections, resulting in the development of diverse cellular and humoral immunological responses [176]. Parallelly, oxidative stress occurs when the equilibrium favors free radical formation over antioxidant components. Inflammation and oxidative stress are linked through a number of signaling mechanisms [27]. Reactive oxygen species (ROS) produced by mitochondria activate a number of transcription factors involved in the production of pro-inflammatory cytokines and mediators (NF-κB, extracellular signal-regulated kinase/ERK, janus kinase/JNK, mitogen-activated protein kinase/MAPK). Similarly, a few cytokines, such as TNF-α and IL-1, can cause mitochondrial ROS generation [177]. Metabolic and cellular alterations are caused by the interaction of ROS with pro-inflammatory cytokines. In other words, the initiation of an uncontrolled inflammatory process in the presence of oxidative stress is a key element in the pathophysiology of chronic diseases like mental, cardiovascular, traumatic, metabolic and autoimmune diseases. Stomatitis, or inflammation of the mouth’s mucous membranes, can cause redness and swelling of the oral tissues as well as prominent and painful ulcers.

Honey has been proposed as an immunologic modulator with two functions: (1) anti-inflammatory activities by downregulating inflammatory transcription factors (NF-κB and MAPK) and/or suppressing the production of pro-inflammatory cytokines, and (2) stimulation of inflammatory mediators such as prostaglandin E2 (PGE2) and cyclooxygenase-2 (COX-2) [178]. Honey is superior to dextromethorphan and diphenhydramine for the treatment of cough-induced upper respiratory infections. Honey is useful against stomatitis because it penetrates fast into the tissues [179,180].

Many studies have found that propolis has anti-inflammatory activities, which may be because of phenolic acids, notably CAPE. Anti-inflammatory properties of propolis are commonly used in mouthwashes. CAPE, in particular, has been proven to have anti-gingivitis properties [181]. Furthermore, in randomized, double-blind, placebo-controlled studies, rinse solutions containing Brazilian green propolis with higher artepillin C concentration decreased gingivitis to same extent as a NaF/cetylpyridinium chloride rinse or a chlorhexidine solution [169]. Royal jelly has been shown to have anti-inflammatory properties [182]. The key anti-inflammatory and immunosuppressive component of RJ that promotes anti-allergic response has been identified as major royal jelly protein 3 [183].

#### 3.2.5. Anti-Cancer Activity of Bee Products in Relation to Oral Health

Oral cancer refers to malignant tumors that develop in mouth, and the majority includes squamous cell carcinoma, also known as mucosal variation. In clinical practice, gingival, jaw, tongue, oropharyngeal, soft and hard sputum, salivary gland, lip, maxillary sinus and face mucosal are all examples of oral cancer [184]. There are several strategies that can combat cancer, but many of them are associated with detrimental side effects on the health of patients As a result, when chemotherapy and radiotherapy are employed systemically or over a large area of tissue to kill malignant cells, they often affect healthy cells as well, resulting in undesirable side effects. Natural anti-proliferative compounds are an excellent alternative. Several recent studies have found that several natural bee products suppress tumor cell growth and spread and induce cancer cell apoptosis [185], implying that these natural chemicals (or their active components) could be used as part of an alternative medical treatment for human tumors.

Honey bee polyphenols have been demonstrated to have improved anti-cancer effects, helping to prevent oral cancer initiation, proliferation and progression. Apoptosis, cell cycle arrest, oxidative stress regulation, inflammation relief, increased mitochondrial outer membrane permeabilization and angiogenesis suppression are all involved in mechanism. Tualang honey inhibited cell proliferation and promoted apoptosis in oral squamous cell cancer under in vitro conditions [186]. The anti-cancer action of crude honey extracts in oral malignancies is most likely due to caspase 3 activation, which induces apoptosis [187]. Purified polyphenols, rather than crude honey, are currently being studied in cancer research. Caffeic acid, phenyl esters, galangin, kaempferol, acacetin, chrysin, quercetin, pinobanksin, apigenin and pinocembrin are simple polyphenols found in honey bees that are prospective pharmacological agents in cancer treatment [187], but cancer of colon, gastric tract, skin, fibrosarcoma and glioma cell have all been shown to be resistant to these phenolic chemicals.

Propolis is an effective antioxidant in the treatment of oral cancer due to presence of high concentration of phenolics and another antioxidant. When glutathione synthesis is low, tumor cells are more vulnerable to radiation impacts [188]. Propolis produces glutathione in hematopoietic tissue, which has anti-cancer properties [189]. Propolis is used as a supplement to help people avoid chronic degenerative disorders like mouth cancer [190]. Propolis contains caffeic acid phenyl ester (CAPE), which possesses anti-mitogenic and anti-cancer characteristics [191]. CAPE could be used as an adjuvant treatment for people with oral squamous cell cancer (OSCC). Because of its high oral absorption and long-term safety profile, propolis is an ideal adjuvant medication for future immunomodulatory or anti-cancer regimens [190]. Flavonoids in propolis stop oral cancer as well as esophagus, stomach, colorectal, prostate and skin cancer [67].

In experimental animals, RJ has shown pharmacological actions, including anti-tumor activity [192]. RJ’s anti-tumor effects were studied using transplantable mice tumors, including advanced leukemia strains and solid tumors [185]. In murine tumor models, effects of RJ formation of tumor and metastasis were investigated [193]. A spontaneous mammary carcinoma (MCa) and a methylcholanthrene-induced fibrosarcoma (FS) of the CBA mouse were employed as transplantable murine tumors. When injected intra-peritoneally or subcutaneously, RJ had no effect on metastases while intravenous administration of tumor cells and RJ dramatically reduced the metastases growth. Royal jelly components like 10-HDA and 4-hydroperoxy-2-decenoic acid ethyl ester possess high anti-proliferative effect.

## 4. Applications of Bee-Based Products in Managing oral Diseases

**Figure 2:**
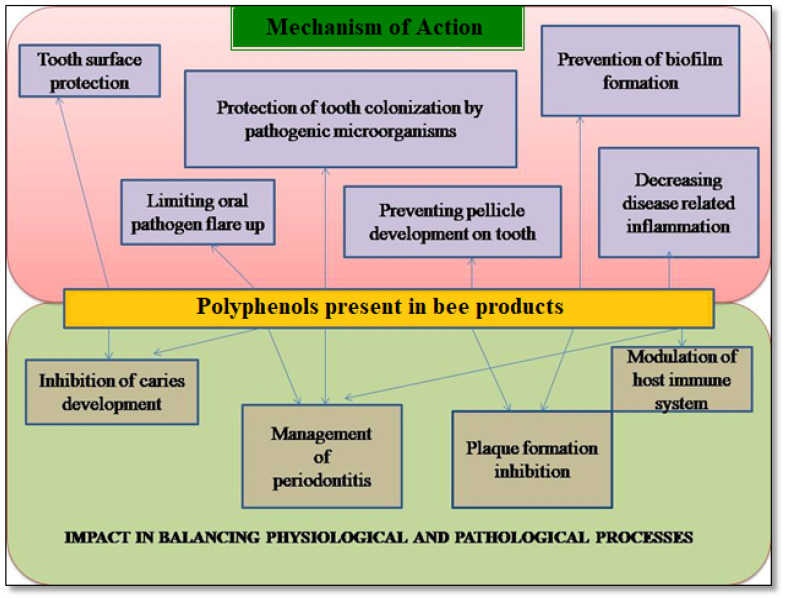
Importance of polyphenols in bee products for oral health.

In recent years, increased implementation of honey bee products like honey, propolis and RJ as an alternative medicine has been witnessed due to their health-promoting activities such as antioxidant, anti-microbial, anti-bacterial, anti-inflammatory, anti-cancer, etc. [25,26,27,28,194]. Honey is employed in treatment of multiple oral ailments such as dental caries, gingivitis, oral cancer, plaque, malodor, radiation-induced oral mucositis and xerostomia [195,196,197]. Similarly, role of propolis has been shown to help with dental caries prevention, oral mucositis reduction, oral cancer prevention and gingival and periodontal disease prevention [37,38,39]. It also reduces dental hypersensitivity and dentin permeability as well as obstruction of dentinal tubules. It works as a transport medium to improve the periodontal ligament cell viability of avulsed teeth as well as for direct pulp capping and analgesia [198,199]. In another study, a positive effect of royal jelly (with anti-microbial activity) on periodontium has been found and that makes it a valuable agent in dentistry [200]. Figure 2 presents the mechanism of action of polyphenols in maintaining oral health.

### 4.1. Gingivitis

Gingivitis arises due to bacterial presence in plaque biofilm resulting in inflammation of gingival tissues. Gingivitis can be treated with good oral hygiene practice, but failing to do so can develop periodontal (gum) disorders leading to loss of alveolar bone and periodontal ligaments attachment [201]. In terms of reducing gingival scores, mouthwash containing manuka honey has been found to be equally efficient as chlorhexidine [202]. The principal infective bacterium responsible for persistent periodontal inflammation is called *Porphyromonas gingivalis*. Levels of inflammatory cytokines, chemokines and CD54 increase in inflammatory sites in periodontal lesions in response to *P. gingivalis* lipopolysaccharides. It has been observed that consumption of royal jelly can inhibit the development of periodontal infections [147].

### 4.2. Dental Caries

The effectiveness of honey has been shown against 60 different species of bacteria, including *S. mutans*, a heavily involved pathogen in dental caries [203]. Presence of bacteria and fermentable carbohydrates along with a susceptible tooth surface are all etiological factors for dental caries. Catechins cause irreversible damage to the microbial cytoplasmic membrane, regulate biofilm production, decrease several cariogenic virulence factors and increase dental caries prevention, according to Xu et al. [204]. Other polyphenols such as flavonols, myricetin and proanthocyanins also work in a similar way by disrupting biofilm development, which inhibit attachment of oral harmful bacteria and reduce acid production by *S. mutans* [205]. Numerous studies have demonstrated anti-bacterial activity of propolis against caries-causing bacteria with no side effects in animal/human models. Fatty acids in propolis act as an anti-caries agent by limiting acid generation and reducing the microorganism’s tolerance to low pH [206]. Hayacibara et al. [207] investigated the impact of propolis on *S. mutans* vulnerability, caries development and glycosyl transferase activity in rats, concluding propolis extract as an anti-caries agent. Nam et al. [208] reported that propolis prevents dental caries by inhibiting the cell division and enzyme activity of bacteria. Propolis has also been incorporated into certain products to prevent caries.

Current research has shown that apigenin and trans-trans farnesol may have biological activity against dental caries by suppressing several virulence associated genes (GtfB and C in *S. mutans*) by apigenin [209]. Topical treatments of 1 mM apigenin, 5 mM tt-farnesol and 13 mM fluoride combination twice a day inhibited the production of *S. mutans* biofilms in the experimental organisms, according to Koo et al. [205]. Likewise, in an in vitro tooth avulsion model, royal jelly solution was observed to be more effective compared to milk and Hank’s balanced salt solution for preservation and transportation [39,210].

### 4.3. Oral Cancer

Oral cancer is among the sixth common type of cancer reported globally [209]. Pathologically, oral squamous cell carcinoma (OSCC) is most widespread contributing remarkably for 84–97% of all cases [211,212]. Tualang honey has been found to exhibit chemopreventive activity due to its phenolic acids and flavonoids in an animal model against 4-nitroquinoline 1-oxide-induced oral cancer [167,213]. Mahmood et al. [214] revealed that *Trigona itama* honey may be an effective chemotherapeutic adjunct in managing HSC-2 cells derived from OSCC. The anti-cancer activity of crude honey extraction in oral cancer appears to be associated with polyphenolic chemical-induced apoptosis via caspase 3 activation. Chrysin, caffeic acid, acacetin, galangin, kaempferol, phenyl esters, pinobanksin, quercetin, pinocembrin and apigenin are the most promising pharmacological compounds identified in honey for cancer treatment. Because of their easy of application in the mouth and established anti-cancer influence on other malignancies, polyphenolic chemicals derived from bee products have a promising future in the creation of a natural, non-toxic, effective alternative in oral cancer treatment [215].

Similar to honey, propolis is found to be one of the natural agents against oral cancer. The anti-cancer properties of propolis are attributed to its flavonoid compounds. Dornelas et al. [216] discovered that pharmacological substances extracted from propolis (artepilin C, p-coumaric acid, CAPE, quercetin, chrysin, caffeic acid and naringenin) had lethal effects in cultured human tumor cells and shrink tumors in animals. CAPE was discovered to suppress oral cancer cell metastasis by modulating matrix metalloproteinase-2 and MAP kinase pathway in another investigation, suggesting that it could be utilized as a chemotherapeutic to prevent oral cancer metastasis [217]. Yanagita et al. [218] reported that royal jelly reduces the production of interleukin 6 and CXC chemokine ligand 10 formation from MPDL22 cells. CD54, a cell adhesion protein involved in the proliferation of leukocytes in periodontal disorders, was also suppressed by royal jelly in MPDL22 cells. These findings suggest that royal jelly has anti-inflammatory and osteoinductive effects, and it may have a role in periodontal disease prevention.

### 4.4. Oral Malodor (Halitosis)

Nowadays, oral malodor (halitosis) draws more attention as it is one of the causes of interpersonal interaction issues. Halitosis can be treated by removing coated tongue with a brushing tooth, tongue scraper, oral prophylaxis and anti-microbial mouth rinse. Patients with oral squamous cell cancer who used manuka honey reported less halitosis, likely due to the honey’s anti-bacterial activity and its ability to divert microbes’ nourishment away from the production of malodor sulfur compounds and toward the production of lactic acid [219,220]. Mouth rinse containing propolis had shown similar effects to those containing essential oils in reducing oral malodor [221].

### 4.5. Oral Mucositis

Honey had been reported to alleviate the severity and duration of radiation-induced oral mucositis (OM), an epithelial injury to the oral, laryngeal or pharyngeal mucosa produced by ionizing radiation during the second and third weeks of radiotherapy [37,222]. Raeessi et al. [223] observed that honey plus coffee regimen is the effective modality for treating oral mucositis. Abdulrhman et al. [37] suggested the honey usage help in faster healing of patients with chemotherapy-induced oral mucositis. RAS Noronha et al. [224] reported that propolis gel with mucoadhesive properties could be used as a potential topical treatment for preventing radiation-induced oral mucositis. Use of propolis assists in replantation of avulsed permanent teeth as well as their healing after oral surgery by reducing inflammation, accelerating the formation of granulation tissue and also imparting analgesic effect [39]. According to Erdem and Güngörmüs [225], royal jelly extract demonstrated a significant reduction in the signs, symptoms and healing time of oral mucositis. Similarly, Yamauchi et al. [226] reported that RJ-treated patients had significantly less mucositis than the non-treated group.

### 4.6. Xerostomia

Xerostomia is defined as a considerable reduction and/or thickening of saliva owing to reduced salivary flow during radiation therapy for neck and head cancer [227]. Charalambous et al. [228] observed that thyme honey is an effective treatment for patients with radiation-induced xerostomia. Thyme honey also enhanced overall quality of life and relieved severe pain and dysphagia. Polyphenolic extracts of honey have been shown to have synergistic effect with antibiotics (e.g., amoxicillin) and are now recommended as a viable alternative to synthetic medications in the prevention and treatment of oral illnesses [229].

### 4.7. Dentin Hypersensitivity

Dentin hypersensitivity (DH) occurs when dentin is exposed to any kind of stimuli such as thermal, osmotic, evaporative, tactile or chemical stimuli. This exposure activates the odontoblast process, resulting in an acute pain [230]. Recently, Tavares et al. [231] reported propolis as an effective, safe and low-cost alternative of reducing DH. Number of researchers [232,233,234,235] have shown evidences that propolis is a promising agent for reducing DH. Current application of bee products and their role in the alleviation of oral pathologies are presented in Table 3.

## 5. Bee Products-Based Innovative Products for Oral Hygiene

Oral and dental and hygiene is practice of maintaining clean and healthy mouth and teeth in order to avoid dental problems such as cavities, gingivitis, periodontal disease and bad breath. Periodontal disorders are caused by poor oral hygiene, which can occur at any age [246,247]. As a result, suitable and effective plaque reduction methods must be implemented on a regular basis [247]. Mechanical approaches, however, may not be practicable or sufficient. As a result, chemical preparations such as anti-bacterial mouthwashes have been proposed as a complement to or replacement for mechanical plaque control [248]. Chlorhexidine (CHX) is most commonly used mouthwash and considered as golden standard against dental plaque [248,249]. However, long-term application of CHX is associated with various adverse effects including changed taste sensitivity, staining of teeth and burning sensation [249,250]. As a result, pharmaceutical companies are working to develop natural-derived oral care products such as toothpaste, mouthwash and chewable tablets or chewing gums based on bee products.

### 5.1. Bee Product-Based Toothpaste

Herbal toothpaste containing clove fruit, neem leaves, honey and Acacia powder for maintaining oral hygiene was developed and it was found that the developed herbal toothpaste was equally effective as per Bureau of Indian standards [251]. The propolis containing toothpaste showed positive biological activity with respect to spectra of oral microbiota without causing adverse effect and can be used as natural alternative to chemical mouthwashes [252]. Similarly, the toothpaste developed using tea tree oil and propolis extract was found to be effective in maintaining oral hygiene by quantitative reduction in oral microbiota due to their anti-microbial and anti-fungal properties [253]. Propolis based toothpaste is intended to prevent the formation of bacterial plaque and pathogenic microflora, which can lead to tooth decay. It has been reported that propolis-based toothpaste was more effective than calcium hydroxide-based toothpaste and showed good anti-bacterial activity [254]. A patent application (CN102283795A) for the toothpaste preparation method with propolis is under consideration. This invention claims effective control and treatment of oral diseases like dental caries by reducing plaque bacteria [255]. Another patent application (CN110755355A) for toothpaste containing propolis extract claims to inhibit pathogenic bacteria in an oral cavity improves bleeding gum and maintain oral health [256].

### 5.2. Bee Product-Based Mouthwash

Mouth rinse has been quite popular due to its ease of use; however, chemical compounds found in mouth rinses such as cetylpyridinium chloride, chlorhexidine and zinc chloride may have side effects if used for long term [257,258]. Propolis-based mouth rinse or mouthwashes have been studied by several researchers and observed great potential in reducing dental plaque and gingival inflammation [259,260,261,262,263]. A patent (CN104739738A) was granted for a mouthwash made with propolis to improve oral health and to treat oral diseases [264].

### 5.3. Bee Product-Based Chewable Tablets or Chewing Gums

Chewable tablets are tablets that are chewed in the oral cavity before being swallowed. These tablets have several benefits such as oral drug delivery without any requirement of water, palatability and stability. These tablets are suitable for children, the elderly and patients suffering from dysphagia. Commercially available chewing gums containing propolis was superior to the one with xylitol gum in reducing bacterial count [265]. A patent (CN107198189B) was granted for a chewable tablet made with propolis, royal jelly, honey, beeswax, sodium citrate, mannitol, maltodextrin and xanthan gum [266]. Another patent application (CN102326723A) is under consideration for development of chewable propolis tablet [267]. Chewing gum are confectionary products that are chewed for various reasons. A worldwide patent (WO2020101601A2) was assigned for producing three types of propolis chewing gums *viz.* sugar, sweetener and sugar-free [268]. Various innovative products developed from the bee products are presented in Table 4.

## 6. Safety Aspects of Honey and Bee Products

Although, consumption of bee products like honey, propolis and royal jelly is safe and allergies and sensitivities to them are uncommon. However, patients who are taking them extensively should be advised about the possibility of adverse reactions and allergies. Toxic chemicals in honey have been reported including polycyclic diterpene grayanotoxins in honey from rhododendron plants such as *R. luteum* and *R. ponticum*. This honey is recognized as “mad honey” because of its severe neural intoxication and even death, particularly in Turkey’s eastern Black Sea region. In spite of its toxicity, it is used as a traditional remedy for sexual dysfunction, hypertension and other diseases, probably because its activity [273,274]. Plants in the Boraginaceae, Asteraceae and Fabaceae families possess pyrrolizidine alkaloids, which are not hazardous but are transformed into injurious pyrrolic metabolites by the liver after ingesting honey. These alkaloids may provide a health danger to honey consumers because they are found in common honey botanical sources [275]. Honey poisoning has been associated to incidences of convulsions, delirium and poor memory due to contamination by the neurotoxic sesquiterpene. Honey bees gather dew produced by passionvine hoppers (*Scolypopa australis*), which feed on sap of the poisonous shrub tutu (*Coriaria spp*.) and swallow these oxygenated sesquiterpene picrotoxanes, which further target GABAergic and glycinergic receptors [276,277]. Hyoscyamine, saponins, strychnine, gelsemine, hyoscine, oleandrin and oleandrigenin are some of the other plant secondary metabolites identified in honey that may be toxic to humans. Honey can be polluted by environmental pollutants such as heavy metals, pesticides and antibiotics in addition to phytochemicals. Furthermore, honey that has been stored or heated for an extended period of time may produce Maillard reaction products [278]. Clinical and in vivo animal studies have shown that propolis is safe and non-toxic. At higher doses (>15 g/day) it shows toxic effects in humans [12]. However, incidences of propolis allergy and contact dermatitis have long been observed, primarily among beekeepers. Furthermore, it has the potential to irritate the skin, causing eczema, lesions, psoriasis and mouth sores [279]. The main sensitizers in propolis, according to Burdock [138] and Walgrave et al. [280], are 3-methyl-2-butenyl caffeate, phenylethyl caffeate, benzyl salicylate, benzyl cinnamate and 1,1-dimethylallylcaffeic acid. Similar to honey, harmful chemicals and pollutants are also present in royal jelly. Pesticides from the organochlorine, organophosphorus and carbamate families are the most common. Eczema, asthma and hypersensitivity have all been linked to royal jelly consumption, and the royal jelly proteins MRJP-1 and MRJP-2 have been recognized as key allergens [281].

## 7. Conclusions

Honey bee products have remarkably high biological and therapeutic properties since they are imparted with a wide variety of bioactive such as phenolic compounds, flavonoids and terpenoids. Studies with in vitro, in vivo and clinical trials showed on anti-microbial activities of bee products against various pathogenic bacteria such as *S. mutans* and *Porphyromonas gingivalis,* antioxidant, anti-inflammatory and anti-cancer against various oral pathologies periodontitis, dental caries, mucositis and dentin hypersensitivity. Many human clinical trials revealed bee products are safe and helpful in the treatment of various oral diseases. It was reported that novel products based on bee products, such as chewing gums, toothpaste, and mouthwash, could be sources of cost-effective and consumer-friendly nutritional components for improving human oral health. However, there is a lot of potential in using these qualities of bee products to cure a variety of diseases. In vitro and intervention study results have been inconsistent in identifying the various functional characteristics of each bioactive compound in bee products, as well as the method to improve their bio-accessibility. Though, honey bee products also cause some side effects like allergies, but they appear only when they are used in high concentrations. The molecular mechanism of action of bee products must be explored in order to fully comprehend their mode of action. In addition, to confirm the influence of bee products on human health, in vivo and human clinical investigations should be conducted. Based on these findings, a policy for using bee products in commercial products must be developed.

## Figures and Tables

**Table 1:** Bioactive compounds with their biological activities in bee products.

Source of Bee Product	Group	Bioactive Compounds	Biological Activity	References
Honey	Flavonoids	flavonoles (^a,b,c,g^ quercetin, ^b,g^ galangin, ^b,f,g^ fisetin, ^b^ myricetin) flavanones (^a,b^ pinocembrin, ^c^ pinobanksin, ^e^ naringenin, ^c,e^ hesperetin) flavones (^c,f^ apigenin, ^b,g^ acacetin, ^b,c,f^ chrysin, ^b,c,e^ Luteolin, ^c^ genistein, ^b^ wagonin) and ^b^ caffeic acid phenethyl ester	^a^ anti-microbial ^b^ anti-cancer ^c^ anti-inflammatory ^d^ anti-fungal ^e^ antioxidant ^f^ anti-bacterial ^g^ anti-allergic ^h^ anti-genotoxic ^i^ neuroprotective ^j^ anti-anxiolytic ^k^ chemoprotective ^l^ anti-proliferative	[61,62,63,64,65]
	Phenolic acid	^h,i^ p-coumaric acid, ^j^ gallic acid, ^e,k,l^ ellagic acid, ^c,e,i^ ferulic acid, ^b,e^ syringic acid		[64]
Propolis	Flavonoids	flavonoles (^a,b,c,g^ quercetin, ^b,g^ galangin, ^b,f,g^ fisetin) flavanones(^a,b^ pinocembrin) flavones (^c,f^ Apigenin, ^b,g^ acacetin, ^b,c,f^ chrysin) and ^b^ caffeic acid phenethyl ester (CAPE)		[61,62,63]
	Phenolic acid	^a^ 2,2-dimethyl-8-phenylchromene, ^a,b,c^ 4-hydroxy-3,5-diprenyl cinnamic acid (artepillin C), ^a^ 3-prenyl cinnamic acid allyl ester, ^b^ kaempferide, ^d^ benzofuran,		[61]
	Terpenoid	^d^ isocupressic acid, ^b^ symphyoreticulic acid, ^a,b,e^ procrim a and b, ^a,b,e^ lupeol, ^d^ farnesol		[61,66]
Royal jelly	Flavonoids	flavonoles (e.g., ^a,b,c,g^ quercetin, ^b^ kaempherol, ^b,g^ galangin and ^b,f,g^ fisetin) flavanones (e.g., ^a,b^ pinocembrin, ^c^ naringin, ^c,e^ hesperidin and isosakuranetin) flavones (e.g., ^c,f^ apigenin, ^b,g^ acacetin, ^b,c,f^ chrysin and ^b,c,e^ luteolin)		[67]
	Phenolic acid	2,2-dimethyl-8-prenylchromene, 3-prenyl cinnamic acid allyl ester, artepillin C		[63]
	Terpenoid	isocupressic acid, labdane diterpenoid		[63]

The bioactive compounds and their associated biological activities are presented in subscripted small letters.

**Table 2:** Biological activities of bee products in relation to oral health.

Type of Bee Product	Type of Extract	Bioactive Compounds	Type of Study	Biological Activity	Key Findings	References
Honey	Aqueous extract	Mixture of phenols and flavonoids	In vitro and in vivo	Antioxidant	In male Wistar rats, application of 1 mg/mL honey showed 45.3% DPPH inhibition. Significant (*p* = 0.028) reduction in lipid per oxidation in oral mucosa wound tissue was observed.	[75]
	Aqueous extract	Flavonoids	In vitro and in vivo	Anti-bacterial	Honey mouth rinse showed antibacterial characteristics and was found effective against oral infections (in vitro). Plaque development was also inhibited (in vivo).	[100]
	Aqueous extract	Phenolic acids, Flavonoids	In vitro	Anti-fungal	The Algerian honeys with different concentrations (undiluted, 10%, 30%, 50% and 70% *w/v*) were tested against *Candida albicans* and *Rhodotorula* sp. Both species had MICs of 70.09–93.48% and 4.90–99.70% *v/v*, respectively.	[101]
	Aqueous extract	Flavonoids	Clinical trial	Antiviral	In children with primary herpetic gingivostomatitis, combining honey with oral acyclovir can yield better results than acyclovir alone.	[102]
	Aqueous extract	Flavonoids	In vitro	Anti-inflammatory	Honey flavonoid extract (HFE) considerably reduce release of pro-inflammatory cytokines such as tumor necrosis factor alpha (TNF-α) and interleukin -1 (IL-1), according to the findings. The formation of reactive oxygen intermediates and the expression of inducible nitric oxide synthase (iNOS) were also dramatically reduced.	[103]
	Aqueous extract	Phenolic acids	In vitro	Anti-cancer	Oral squamous cell carcinoma (OSCC) and osteosarcoma (HOS) cell lines showed maximum suppression of cell growth of 80% at a concentration of 15%.	[104]
Propolis	Aqueous extract	Flavonoids	Clinical trial	Antioxidant and anti-inflammatory	Propolis reduced and delayed radiation-induced mucositis in rats by being able to prevent reduction in salivary antioxidant levels.	[91]
	3% ethanolic extract (EEP)	Flavonoids:—kaempferol and quercetin and cinnamic acid derivatives	Clinical trial	Anti-bacterial	At 50 mg/L, EEP had a time-dependent microbiological effect with anti-bacterial efficacy against Gram-positive bacteria. Hygienic treatments containing 3% EEP effectively assist plaque clearance and enhance the condition of the marginal periodontium.	[105]
	70% ethanol	Esters of phenolic acids (caffeates and ferulates) and flavonoids	In vitro	Anti-bacterial, Anti-fungal and Antiviral	All the studied samples exhibited significant activity against fungal and Gram-positive bacterial test strains, with the majority also showing antiviral activity.	[106]
	Aqueous and ethanol extracts	Galangin and chrysin	In vitro	Antiviral	In viral suspension experiments, both propolis extracts were found to have significant antiviral efficacy against HSV-1, with plaque formation reduced by >98%.	[107]
	Aqueous extracts	Artepillin C	Clinical trial	Anti-inflammatory	Rinse products containing Brazilian green propolis high in artepillin C reduced gingivitis to the same extent as a NaF/cetylpyridinium chloride rinse or a chlorhexidine solution in randomized, double-blind, placebo-controlled studies.	[108]
	Ethanol extracts	Caffeic acid phenethyl ester	In vitro	Anti-cancer	In TW2.6 human oral squamous cell carcinoma (OSCC) cells, propolis extracted caffeic acid phenethyl ester CAPE treatment reduced cell proliferation and colony formation in a dose-dependent manner. CAPE treatment reduced the number of G1 phase cells, increased the number of G2/M phase cells, and caused death in TW2.6 cells.	[109]
Royal Jelly	Aqueous extracts	I-IV jellein peptides	In vitro	Anti-bacterial	Four peptides were isolated from honey bee and Royal Jelly presented exclusively antimicrobial activities against Gram-positive and Gram-negative bacteria.	[29]
	Aqueous extracts	Phenolic compounds	In vitro	Anti-fungal	The MIC, MIC50 and MFC of Royal Jelly on Candida albicans were 80, 103 and 160 mg/mL, respectively, while the MIC, MIC50 and MFC of Iranian Propolis alcoholic extract were 0.030, 0.0618 and 0.0833 mg/mL, respectively.	[110]
	Aqueous extracts	Major royal jelly protein 3 (MRJP3)	Clinical trial	Anti-inflammatory	When RJ suspension was given to a culture of mouse peritoneal macrophages activated with lipopolysaccharide and IFN-gamma, the production of proinflammatory cytokines such as TNF-α, IL-6 and IL-1 was efficiently reduced in a dose-dependent manner without causing macrophage cytotoxicity.	[111]
	Aqueous extracts	10-Hydroxy-2-decenoic acid	In vitro	Anti-cancer	The 10-HDA at 20 M or higher significantly suppressed proliferation and migration of cancerous cells.	[112]

**Table 3:** Current applications of bee-based products in the improvement of oral health.

Type of Bee Product	Disease Targeted	Whole Bee Product/Extracts and Dose	Key Findings	Reference
Honey	Radiotherapy-induced xerostomia	Individuals suffering from neck and head cancer were randomly allocated to the control group (oral rinses with saline) and the intervention group (oral rinses with 20 mL of thyme honey diluted in 100 mL of purified water). Patients were required to perform oral rinses just before, immediately after and 6 h after the radiotherapy session. Radiation-induced xerostomia was assessed starting from the 4th week of radiotherapy, one and six months after the completion of radiotherapy	Thyme honey was found useful in lowering or stabilizing the degree of xerostomia over time, with progressive improvement. The better management of xerostomia showed significant effects on overall quality of life.	[228]
	Chemotherapy-induced mucositis	Patients randomly assigned to one of three treatment groups: Group 1—Empirical dosage of 0.5 g honey/kg (max.15 g) was applied topically three times daily to the afflicted oral mucosa for 10 days, or until healing. Group 2—An empirical dose of 0.25 g/kg (max. 5 g) of a 4:2:1 mixture of honey, olive oil-propolis extract, and beeswax (HOPE) administered topically three times daily to the diseased oral mucosa for 10 days, or until healing. Group 3—Served as the control group where benzocaine 7.5% gel was topically applied three times daily to the afflicted oral mucosa.	Honey (Group 1) resulted in faster healing than HOPE (Group 2) or control (Group 3) in both grades of mucositis (*p* > 0.05).	[37]
	Dental Plaque	At the commencement of the trial, each patient underwent a professional prophylaxis to completely remove plaque and calculus from the teeth. The patients were randomly assigned to below groups: Group 1—Manuka honey was applied gently to gingival sulcus of the teeth, and the procedure was repeated twice after 5 min. The honey was applied twice a day after meals. Group 2—Rinsing with 0.2% chlorhexidine (10 mL) twice a day for 60 s followed by its expectoration. Group 3—Chewing the xylitol chewing gum for 5 min, thrice a day after meals. Following the experimental time, the plaque scores were determined.	The mean plaque scores for Groups 1, 2 and 3 were correspondingly 1.37, 1.35 and 1.57. The study indicated that manuka honey and chlorhexidine mouthwash significantly reduced plaque development compared to xylitol chewing gum.	[236]
	Dental Plaque	Dental plaque score was recorded in individuals before and after gargling with Tongra original honey 5% solution for six days.	Gargling with Tongra original honey 5% solution effectively decreased the dental plaque score.	[237]
	Oral squamous cell carcinoma (OSCC) and human osteosarcoma (HOS)	Different Tualang honey (1–20%) concentrations were administered to OSCC and HOS cell lines at different intervals of 3, 6, 12, 24, 48 and 72 h.	Tualang honey had an anti-proliferative effect on both cell lines. Maximum inhibition (≥80%) of cell growth recorded at a dose of 15%.	[238]
	Oral mucosal ulcers	For the oral mucosal ulcer model, excisional wounds were conducted on 30 Wistar albino rats (240–30 g) and they were separated into 3 groups: Group 1—Apitherapeutic agent or honey treatment (0.1 ml, 2 × 1). Group 2—Glyceroloxytriester (TGO) (0.1 ml, 2 × 1) was used to treat locally. Control Group 3—On the 7th day, biopsy samples were collected from the right buccal mucosa, and on the 14th day, samples were taken from the left buccal mucosa.	Only on the 7th day a significant difference documented between groups 1 and 3, whereas on day 14, no significant difference was noted among the groups. Honey was found to be efficient in the therapy of oral mucosal ulcers and showed a greater therapeutic benefit than glyceroloxytriester (TGO).	[239]
Propolis	Gingivitis	One of the twins got 2% pure propolis for rinsing during the gingivitis induction period while the other received a color-matched 0.05% sodium fluoride + 0.05% cetylpyridinium chloride for rinsing (positive control). For 21 days, patients were advised to rinse 20 mL of respective rinses twice a day for 30 s each time.	During a 3-week no-hygiene period, a 2% typified propolis rinse performed similar to positive control rinse.	[240]
	Radiotherapy-induced mucositis	Patients were divided into two groups at random: The Case group (n = 10) received 15 mL of water-based propolis mouthwash three times daily, while the Control group (n = 10) received 15 mL of placebo mouthwash.	Propolis water extract effectively prevented and cured radiotherapy-induced mucositis.	[241]
	Denture stomatitis	The patients were randomized to one of two therapy groups at random: Miconazole oral gel, 20 mg/g, was given to the control group (MIC) for 14 days. For 14 days, the PROP group was given a mucoadhesive formulation containing a standardized extract of 2% (20 mg/g) propolis (EPP-AF ^®®^). On days 1, 7 and 14, patients were assessed.	EPP-AF showed effect at par with miconazole.	[242]
	Cariogenic infections in a caries-active patient	Patients were randomly assigned to one of three experimental groups after their cavitated lesions were restored: (1) PROP-alcohol-free 2% propolis rinse (n = 20); (2) CHX- 0.12% chlorhexidine rinse; (3) PL-placebo mouth rinse. Patients were asked to rinse with 15 mL of respective rinses twice day for 60 s for 28 days. Salivary levels of Mutans *Septococci* (MS) and *Lactobacilli* (LACT) were evaluated at baseline, 7-day, 14-day and 28-day visits (experimental effects) and 45-day visits (residual effects).	Among all the treatments evaluated, propolis rinse was found to be the most efficient at suppressing cariogenic infections in caries-active participants.	[243]
	Dental Plaque and Gingivitis	A phase II clinical trial involved patients with a minimum of 20 healthy natural teeth, a mean plaque index of at least 1.5 P I, and a mean gingival index of at least 1.0 GI. Patients were advised to rinse with 10 mL of mouthwash test for one minute after brushing their teeth in the morning and at night.	Plaque and gingival index considerably reduced after 45 and 90 days of using mouthwash.	[244]
	Dentin hypersensitivity (DH)	96 patients with DH in one or more teeth were studied in this study. The teeth were allocated in one of four treatment groups at random: Group 1—10% ethanolic extract of propolis. Group 2—30% ethanolic extract of propolis. Group 3—Single Bond Universal dentin bonding agent. Group 4—Distilled water as a placebo. The degree of DH was assessed using a visual analog scale based on the patients’ response to tactile and air blast stimuli.	Propolis extracts and dentin bonding agent were found to be equally effective in relieving DH. Propolis application was advised for patients experiencing mild to moderate discomfort whereas dentin bonding agent could be a preferable alternative for rapid relief.	[234]
	Oral malodor	Patients were allocated into three groups: I received a placebo (P), II received an ethanolic extract of propolis type-3 at a concentration of 3% (EEP), and III received chlorhexidine (CHX) at a concentration of 0.12%. Participants were instructed to rinse with respective rinses twice a day for 5 days. Each trial period was followed by a washout period of 21 days. Microbiological samples were obtained from the tongue dorsum at baseline and at the end of the rinse period to measure the concentration of volatile sulfur compounds (VSC) in the morning mouth breath.	Both EEP and CHX treatment resulted in a considerable reduction in the mean counts of bacterial pathogens, including certain VSC producers. The study suggested the use of mouth rinse containing 3% propolis type-3 prevents malodorous morning breath.	[245]
Royal Jelly	Periodontal pathogens	Subgingival plaque samples were obtained and analyzed from 15 chronic periodontitis patients. RJ and chlorhexidine were tested in vitro for their ability to inhibit the growth of aerobic and anaerobic bacteria.	Chlorhexidine at low dose (50 μg/100 μL) was shown to be more sensitive in suppressing aerobic and anaerobic periodontopathic bacteria in subgingival plaque area whereas a larger dosage of RJ was required to have an inhibitory effect.	[150]
	Radiotherapy-induced mucositis	Patients who needed chemoradiation for head and neck cancer were randomly allocated to one of two groups. During chemoradiation treatment, seven patients in the experimental group received RJ three times a day while six patients in the control group did not. The development of mucositis in both groups of patients was monitored twice a week.	The use of RJs reduced radiation-induced mucositis in patients with head and neck cancer.	[226]
	Radiotherapy and chemotherapy-induced mucositis	Patients were allocated into two categories. Both groups used benzydamine hydrochloride and nystatin mouthwashes. The experimental group also received 1 g RJ twice daily in addition to mouthwash. Both experimental groups administered mouthwash and/or RJ to patients until the mucositis resolved.	The RJ group resolved oral mucositis in a fraction of the time it took the control group.	[225]

**Table 4:** Various innovative products developed from bee products.

Type of Product	Product	Patents	Bee Product Used	Intended Use	Reference
Toothpaste	Propolis toothpaste	CN102283795A	Propolis	To prevent and treat oral diseases	[255]
	Propolis toothpaste	CN110755355A	Propolis	To improve bleeding gum and maintain oral health by inhibiting pathogenic bacteria	[256]
	Brazilian green propolis toothpaste	CN107412138B	Propolis	To prevent on gingivitis, periodontitis and halitosis	[269]
	Manuka honey toothpaste	CN105287328A	Manuka honey	To prevent prevents gingivitis, periodontitis, tooth decay and oral ulcer during pregnancy	[270]
Mouthwash	Propolis mouthwash	CN104739738A	Propolis	Improved anti-bacterial and anti-inflammatory properties, as well as the prevention and treatment of oral disorders such oral cancer	[264]
	Propolis mouthwash	KR20060041348A	Propolis	To prevent tooth decay and treat oral diseases	[271]
Chewing gum	Hive honey chewing gum	CN107751533A	Honey	To improve health of oral cavity and remove bad breath	[272]
Chewable tablet	Tablet	CN107198189B	Propolis, royal jelly, honey, and beeswax	To improve immunity and highly suitable for patients suffering from pharyngitis	[266]
